# HBV suppresses expression of MICA/B on hepatoma cells through up-regulation of transcription factors GATA2 and GATA3 to escape from NK cell surveillance

**DOI:** 10.18632/oncotarget.11271

**Published:** 2016-08-12

**Authors:** Yun Guan, Weiqun Li, Zhaohua Hou, Qiuju Han, Peixiang Lan, Jian Zhang, Zhigang Tian, Cai Zhang

**Affiliations:** ^1^ Institute of Immunopharmacology and Immunotherapy, School of Pharmaceutical Sciences, Shandong University, Jinan, Shandong, China; ^2^ Institute of Immunology, School of Life Sciences, University of Science and Technology of China, Hefei, Anhui, China

**Keywords:** hepatitis B virus, MICA, hepatocarcinoma, GATA-2, GATA-3, Immunology and Microbiology Section, Immune response, Immunity

## Abstract

Decreased expression of NKG2D ligands on HBV-infected human hepatoma cells impairs NK cells lysis. However, which components of HBV exert this effect and the precise mechanisms need to be further investigated. In the present study, we observed that the HBx and HBc genes significantly down-regulated MICA expression. Through analysis with the chromatin immunoprecipitation assay, we found that HBV infection promotes the expression of transcription factors GATA-2 and GATA-3, which specifically suppressed MICA/B expression by directly binding to the promoter region of MICA/B. HBx protein, acting as a co-regulator, forms a tripolymer with GATA2 and GATA3, thus promotes the GATA-2 or GATA-3-mediated of MICA/B suppression. HBc protein inhibits MICA/B expression via directly binding to the CpG island in the MICA/B promoter. Thus, our study identified the novel role of transcription factors GATA-2 and GATA-3 in suppressing MICA/B expression and clarified the mechanisms of HBx and HBc in downregulation of MICA/B expression. These findings provide novel mechanisms for the contribution of HBV to hepatoma cells escape from NK cell surveillance.

## INTRODUCTION

Natural killer (NK) cells represent the main effector population of the innate immune system in the defense against virus infection and tumors [1, 2]. NK cells express a variety of activating and inhibitory receptors which control the function of NK cells [3]. As a potent activating receptor, the NKG2D receptor plays an important role in the control of viral infections and tumorigenesis through recognizing its ligands, including MICA, MICB and ULBP1-4 in humans and Rae-1, H60 and MULT1 in mice [4]. However, viruses have evolved various mechanisms to counteract NKG2D-dependent immune responses. For example, MCMV m152-encoded gp40 decreases the surface expression of H60 and Rae-1 to inhibit NK cell cytotoxicity. HCMV-encoded soluble proteins, UL16 and UL142, markedly reduce cell surface levels of NKG2D ligands and compromise the efficacy of NK cell responses [5]. Thus, a better understanding of the regulatory mechanism of NKG2D and its ligands during virus infection and tumorigenesis is needed.

Hepatitis B virus (HBV) infection is one of the main causes of chronic liver diseases including liver cirrhosis and hepatocellular carcinoma. genome consists of four open reading frames (ORF), namely X, C, S and P, encodes HBx, HBc protein, HBs antigen, HBe antigen and DNA polymerase [6, 7]. These HBV proteins constitute and assemble to HBV particles, moreover, they are involved in regulating viral and host gene expression. For example, the HBx protein is a multifunctional regulator that modulates gene transcription, signal transduction, cell cycle progress and epigenetic modifications [8]. HBc protein can modulate gene expression by binding to a large number of gene promoters in human genome and CpG islands of HBV cccDNA [9, 10].

Although NK cells play a critical role in the clearance of HBV, HBV infection may escape the surveillance of NK cells by altering the activation status and receptor expression patterns on the surface of NK cells [11]. For example, the expression of inhibitory receptors NKG2A is elevated, while activating receptors (*e.g*. CD16, NKG2D and NKp30) are downregulated during HBV infection [3]. Impairment of NK cell activation and function may also arise from modified expression patterns of ligands for NK cell receptors. Indeed, decreased expression of the NKG2D ligands, MICA/B, on HBV-infected human hepatoma cells has been shown to depress NK cells lysis [12, 13]. However, which HBV components impair NK cell anti-viral activity and the exact mechanisms need to be further investigated.

In the present study, we demonstrated that the HBx and HBc could suppress the expression of MICA in hepatoma cells, thus leading to decreased susceptibility of HBV^+^ hepatoma cells to NK lysis. Moreover, we found for the first time that the transcription factors GATA-2 and GATA-3 could bind to the MICA promoter to suppress its expression. The HBx protein, acting as a co-regulator, could bind to GATA-2 and GATA-3 to form a tripolymer and contribute to the down-regulation of MICA expression mediated by these transcription factors. The HBc protein was found to bind to the CpG island of the MICA and MICB promoter to suppress their expression.

## RESULTS

### HBV^+^ hepatoma cells express lower levels of MICA/B, ULBP1, 2, 3 and are less susceptible to NK lysis than HBV^−^ hepatoma cells

**Figure 1:**
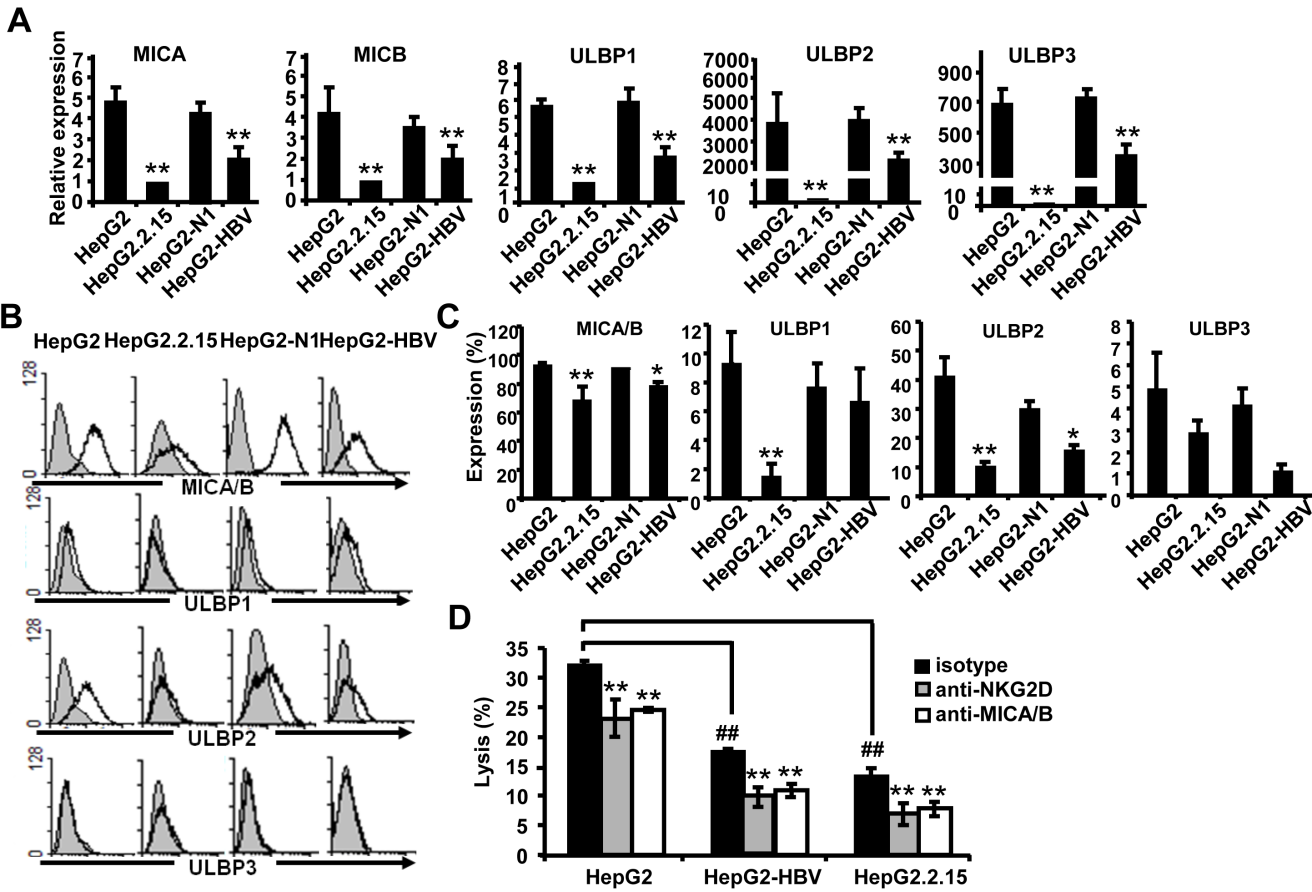
HBV^**+**^ hepatoma cells express lower levels of NKG2D ligands and are less susceptible to NK lysis than HBV^**-**^ hepatoma cells **A.** Expression levels of MICA, MICB, ULBP1, ULBP2 and ULBP3 on HepG2, HepG2.2.15, HepG2-N and HepG2-HBV cells were analyzed by qPCR. **B.** Flow cytometric analysis of NKG2D ligands expressed on HepG2, HepG2.2.15, HepG2-N and HepG2-HBV cells. **C.** Statistical analysis of NKG2D expression on HepG2, HepG2.2.15, HepG2-N and HepG2-HBV cells with FACS. **D.** HepG2, HepG2-HBV and HepG2.2.15 cells were incubated with isotype (Ctrl) or blocking antibody against MICA/B at 37°C for 2 h. At the same time, NKL cells were incubated with isotype (Ctrl) or blocking antibody against NKG2D at 37°C for 2 h. The treated hepatoma cells and NKL cells were then co-incubated for 6 h, and the cytotoxicity of NK cells was measured by the CFSE/7AAD assay. **P < 0.01; *P < 0.05, compared with HepG2, HepG2-N cells or isotype control with paired *t*-test. ##*P* < 0.01, compared with HepG2 cells (paired *t*-test).

HBV has been reported to down-regulate the MICA expression on HepG2.2.15 cells compared with HepG2 cells [12]. We explored the regulatory role of HBV infection in the expression of NKG2D ligands, including MICA, MICB, ULBP1, ULBP2 and ULBP3, on HepG2, HepG2.2.15, HepG2-N and HepG2-HBV cells (Figure 1A-1C). Expression levels of all NKG2D ligands, especially MICA/B and ULBP2, were found to be decreased on HBV-positive HBV-HepG2 and HepG2.2.15 cells at both the gene and protein levels, compared with HBV-negative HepG2 and HepG2-N cells. The interactions of NKG2D and its ligands are known very important for promotion NK cell activation and cytolysis. Therefore, we compared the susceptibility of these HBV^+^ and HBV^−^ hepatoma cells to NK lysis by using the CFSE/7AAD method. The NKL human NK cell line was used as effector cells. As shown in Figure 1D, the cytotoxicity of NKL cells against HBV^+^ HepG2.2.15 and HepG2-HBV was much lower than that against HBV^−^ HepG2 cells, suggesting that HBV decreased the susceptibility of hepatoma cells to NK cytolysis. After blocking MICA/B on hepatoma cells with an anti-MICA/B antibody or blocking of NKG2D on NKL cells with an anti-NKG2D antibody, the cytolytic activity of NKL cells decreased significantly. These results suggest that the lytic activity of NKL against hepatoma cells was correlated with the interaction between NKG2D and its ligands. HBV infection could reduce the susceptibility of hepatoma cells to NK lysis possibly by down-regulation of NKG2D ligands. We also detected the changes of other NK lysis-related molecules, such as MHC class I molecules HLA-ABC, inhibitory ligand PD-L1 and the cell apoptosis-related molecule Fas. The data revealed that HBV could reduce Fas expression while inducing PD-L1 expression, which may also contribute to the reduction of NK cytolysis (Supplementary Figure S1).

### HBx and HBc suppress MICA and MICB expression in hepatoma cells

**Figure 2:**
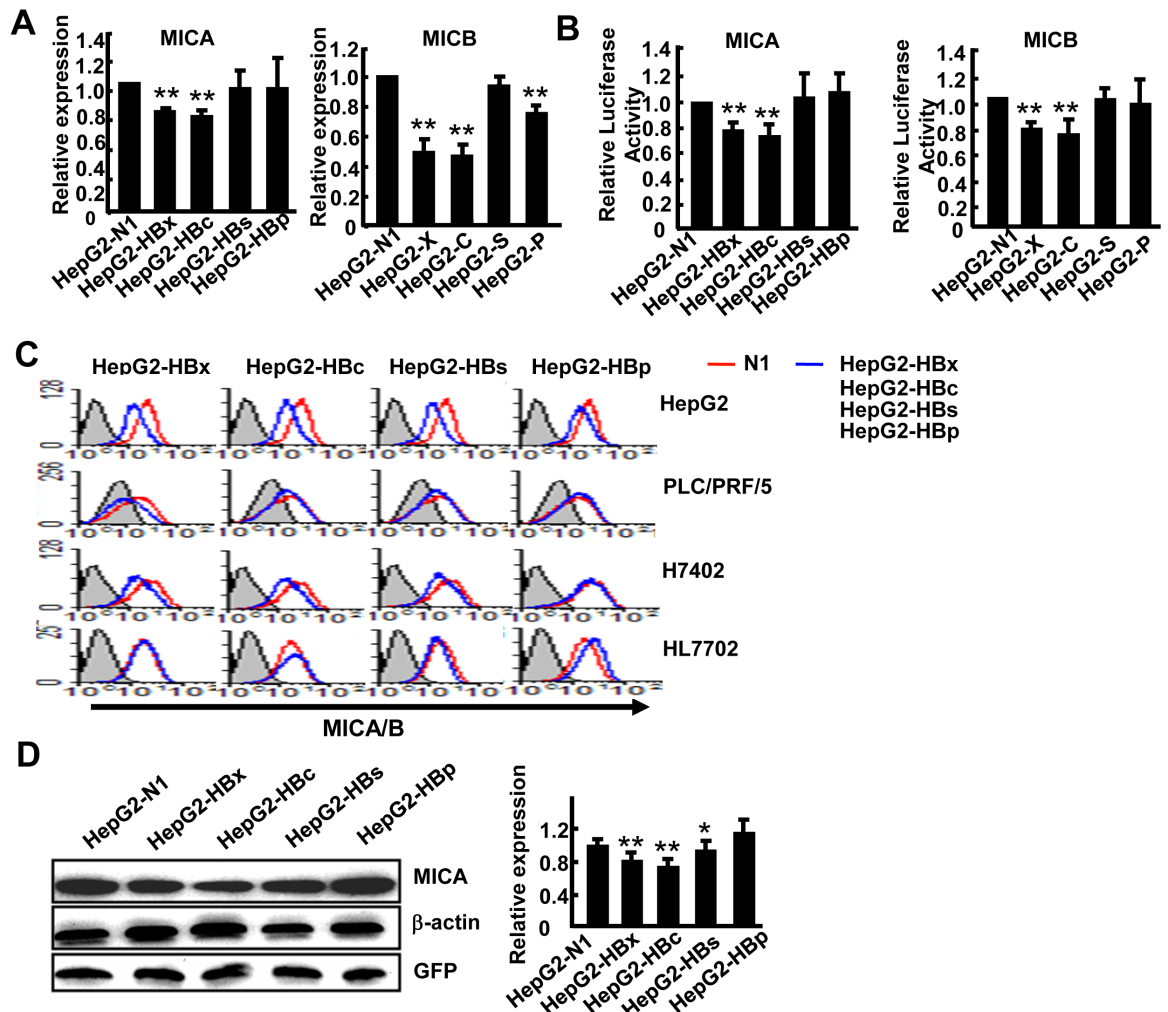
Overexpression of HBx and HBc genes suppresses MICA expression in hepatoma cells **A.** pEGFP-N1, pEGFP-HBx, pEGFP-HBc, pEGFP-HBs or pEGFP-HBp was transfected into HepG2 cells, and 48 h later MICA and MICB expression levels were analyzed by RT (reverse transcription) -PCR. **B.** HepG2 cells were transfected with a reporter plasmid containing the MICA or MICB promoter together with pEGFP-N1, pEGFP-HBx, pEGFP-HBc, pEGFP-HBs or pEGFP-HBp for 36 h. Renilla luciferase activity was normalized to firefly luciferase activity. **C.** HepG2, PLC/PRF/5, H7402 and HL7702 cells were transfected with pEGFP-N1, pEGFP-HBx, pEGFP-HBc, pEGFP-HBs or pEGFP-HBp for 48 h, and then MICA/B expression was analyzed by flow cytometry. **D.** Western blot analysis of MICA expression in HepG2 cells after transfection with pEGFP-N1, pEGFP-HBx, pEGFP-HBc, pEGFP-HBs or pEGFP-HBp for 48 h. The densitometry analysis of MICA expression normalized to GFP. **P < 0.01; *P < 0.05, compared with HepG2-N1 (paired *t*-test).

As described above, HBV downregulated the expression of NKG2D ligands on hepatoma cells. The HBV genome contains four genes (HBx, HBs, HBc, HBp) which encode corresponding proteins to generate diverse biological effects. To investigate which gene regulates the expression of these NKG2D ligands, we amplified the complete ORFs of the four genes from HepG2.2.15 cDNA and separately cloned them into the pEGFP-N1 eukaryotic expression vector. The four resulting vectors, pEGFP-N1-HBx, pEGFP-N1-HBc, pEGFP-N1-HBs and pEGFP-N1-HBp, were confirmed to successfully express HBx, HBV core protein, HBs and HBV polymerase separately [14, 15]. We transfected each of these four vectors into HepG2 cells. Because levels of ULBP1 and ULBP3 were very low in HepG2 cells, we only detected the expression MICA/B and ULBP2. The results showed that transfection with HBx and HBc genes obviously reduced MICA and ULBP2 expression levels, while HBx, HBc and HBp decreased MICB expression (Figure 2A and Supplementary Figure S2). As the expression of MICA/B was much higher than that of ULBP2 on HepG2 cells, we focused on MICA/B expression in the subsequent experiments. We constructed luciferase reporter gene vectors under the control of the MICA and MICB promoter separately to observe the effects of HBV genes on regulation of the transcriptional activity of MICA or MICB. The results demonstrated that the HBx and HBc genes significantly decreased the promoter activity of both MICA and MICB, while other genes did not show any effect (Figure 2B). We further confirm the down-regulatory effect of HBx and HBc on MICA/B expression at protein levels on HepG2 cells and other HCC cell lines (PLC/PRF/5 and H7402) by FACS. As described in Figure 2C, transfection with HBx and HBc genes indeed most markedly reduced the expression of MICA/B, while none of the HBV genes had any influence on MICA/B expression in the normal hepatocytes cell line HL7702. Due to the low level expression of MICB (Supplementary Figure S3A), we further detect MICA protein expression by western blotting with anti-MICA antibody and revealed similar results (Figure 2D).

**Figure 3:**
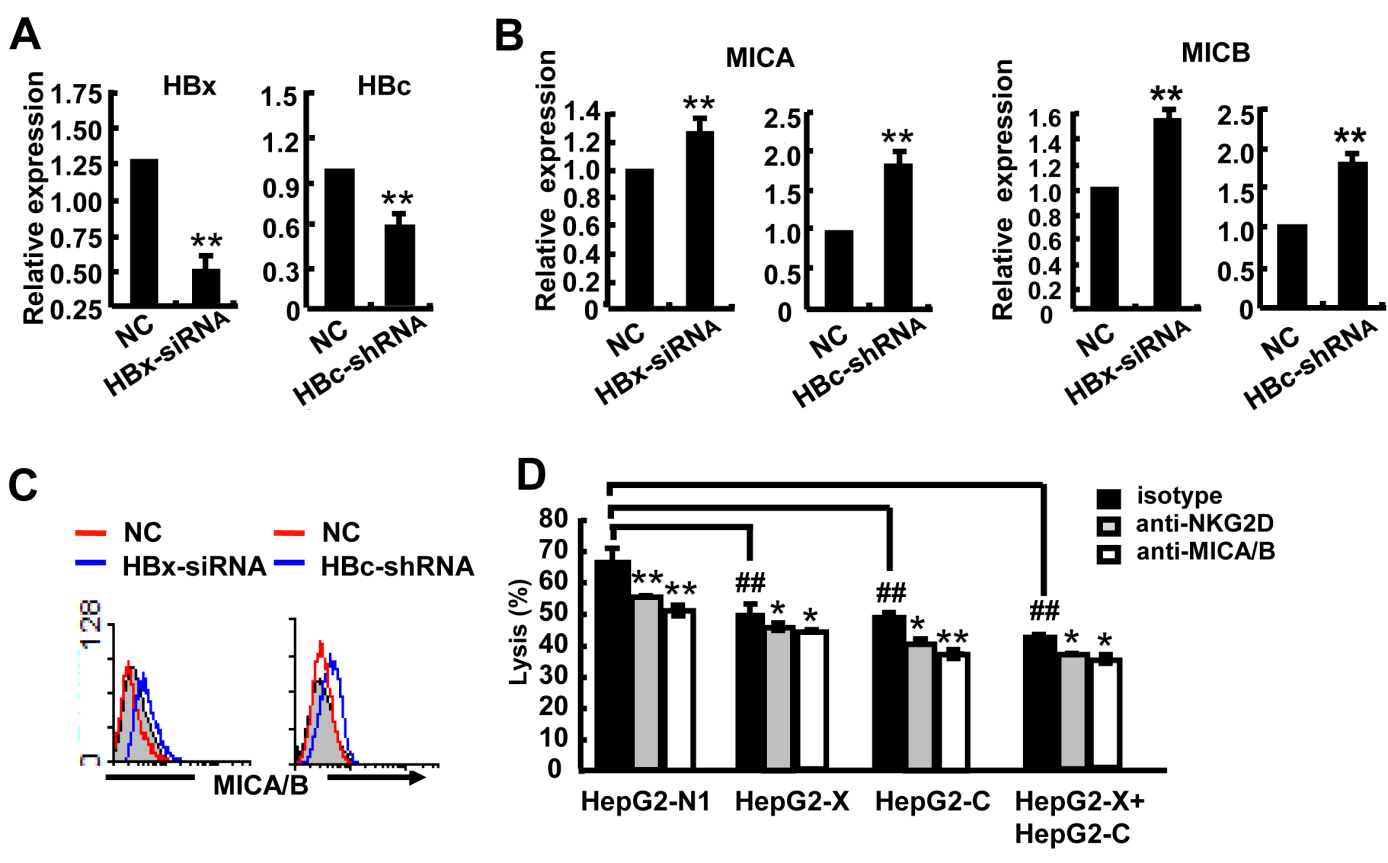
HBx and HBc reduce the susceptibility of HepG2 cells to NK lysis **A.**-**C.** HBx-siRNA or HBc-shRNA was transfected into HepG2.2.15 cells for 24 h, and then MICA expression was detected by qPCR and FACS. **D.** HepG2 cells were transfected with pEGFP-N1, pEGFP-HBx, pEGFP-HBc and pEGFP-HBx plus pEGFP-HBc for 48 h and incubated with blocking MICA/B antibody at 37°C for 2 h. NKL cells were incubated with blocking NKG2D antibody at 37°C for 2 h. The treated hepatoma cells and NKL cells were co-incubated for 6 h, and the cytotoxicity of NK cells was measured by the CFSE/7AAD assay. **P < 0.01; *P < 0.05, compared with negative control (paired *t*-test).

To further confirm the role of HBx and HBc genes in the regulation of MICA/B expression, we transfected HBx-siRNA or HBc-shRNA into HepG2.2.15 cells (Figure 3A) and found increased expression of MICA and MICB at both the mRNA and protein levels (Figure 3B, 3C). This data further confirmed that the HBx and HBc genes could suppress the expression of MICA/B in hepatoma cells. As expected, transfection with HBx and HBc significantly decreased the susceptibility of HepG2 cells to NK lysis, while co-transfection with both HBx and HBc showed much more significant effect. Blocking MICA/B on HepG2 cells or NKG2D on NK cells further attenuated the cytotoxicity of NK cells (Figure 3D). These data demonstrated that the HBx and HBc genes down-regulated the expression of MICA and MICB and further reduced the susceptibility of HepG2 cells to NK lysis.

### HBV infection promotes expression of transcription factors GATA-2 and GATA-3, which specifically suppress MICA/B expression

**Figure 4:**
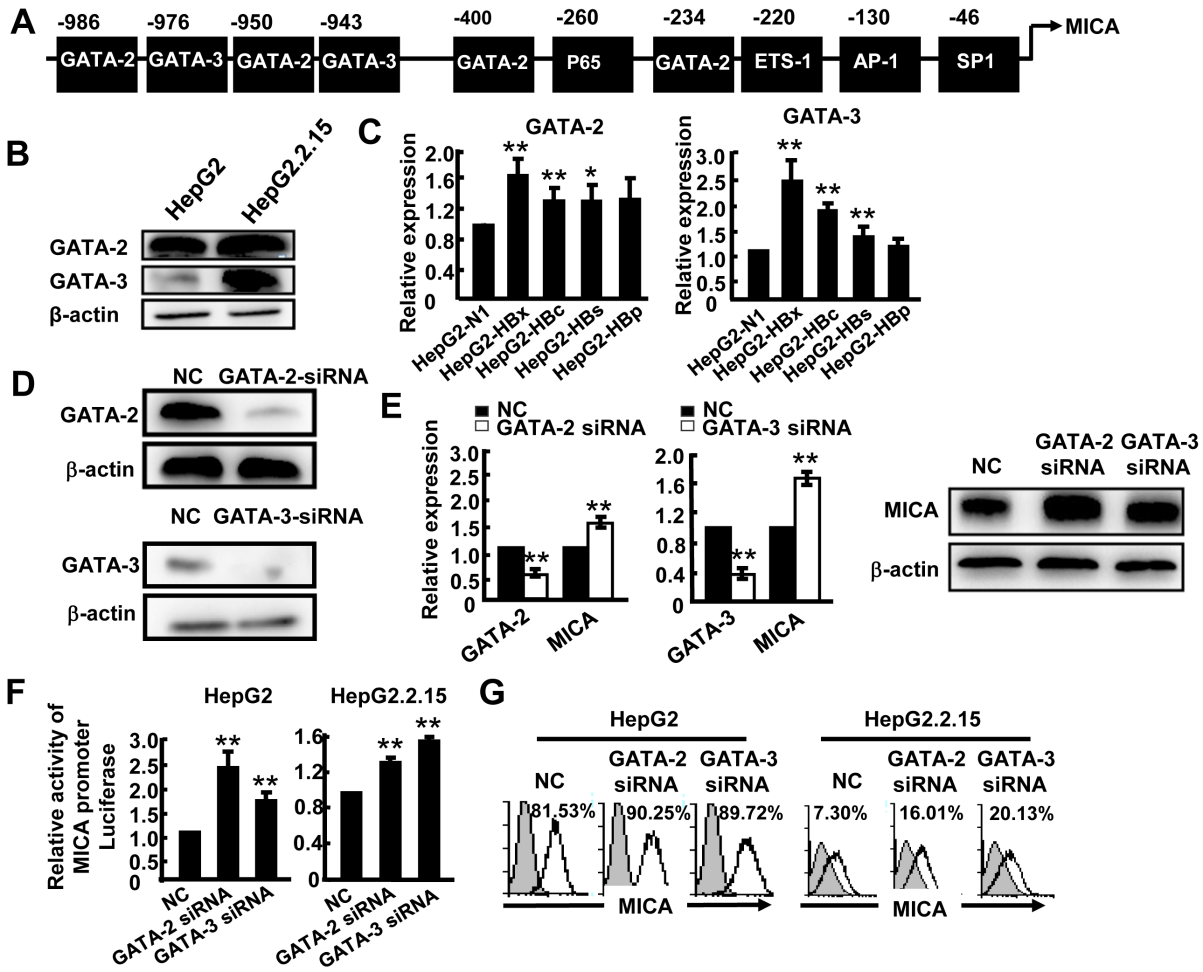
GATA-2 and GATA-3 suppress MICA expression **A.** Prediction of GATA-2 and GATA-3 binding sites in the MICA promoter. **B.** Western blot analysis of GATA-2 or GATA-3 expression in HepG2 and HepG2.2.15 cells. **C.** HepG2 cells were transfected with pEGFP-N1, pEGFP-HBx, pEGFP-HBc, pEGFP-HBs or pEGFP-HBp for 48 h, and then GATA-2 and GATA-3 mRNA levels were detected by qPCR. **D.** GATA-2-siRNA or GATA-3-siRNA was transfected into HepG2.2.15 cells for 48 h. The silence effect was detected by Western blot. **E.** GATA-2, GATA-3 and MICA expression was measured by qPCR (left) and Western blot (right). **F.** HepG2 cells or HepG2.2.15 cells were transfected with GATA-2-siRNA or GATA-3-siRNA together with a MICA promoter reporter plasmid for 36 h, and then relative luciferase activity was examined. Renilla luciferase activity was normalized to firefly luciferase activity. **G.** Flow cytometric analysis of MICA expression in HepG2 or HepG2.2.15 cells after transfection with GATA-2-siRNA or GATA-3-siRNA. **P < 0.01; *P < 0.05, compared with negative control (paired *t*-test).

We next tested the hypothesis that the HBx and HBc genes may influence the levels of some certain transcription factors to regulate MICA/B expression. Transcription factor binding sites analysis tools (JASPAR database and TFSEARCH) were used to predict the most likely transcription factors binding to the MICA or MICB promoter. As shown in Figure 4A, in addition to AP-1, SP-1 and NF-κB, which have been reported to be involved in the regulation of MICA expression [16, 17], four GATA-2 and two GATA-3 binding sites were found at the MICA promoter region (Figure 4A); meanwhile, one GATA-2 and one GATA-3 binding site were observed at the MICB promoter (Supplementary Figure S3B). GATA-2 has emerged as a candidate regulator of gene expression in hematopoietic cells and nonhematopoietic embryonic stem cells [18]. GATA-3 is a main regulator of T cell development and plays a crucial role in endothelial cell biology [19]. However, whether GATA-2 and GATA-3 are involved in MICA/B expression in hepatoma cells is still unknown. First, we examined the GATA-2 and GATA-3 expression levels by Western blotting and found that they were both significantly higher in HepG2.2.15 cells than in HepG2 cells (Figure 4B), suggesting that HBV infection increased the expression of these transcription factors. Furthermore, we tested GATA-2 and GATA-3 expression in HepG2 cells which were separately transfected with four HBV genes (HBx, HBs, HBc and HBp). The results showed that HBx, HBc and HBs genes could markedly increase GATA-2 and GATA-3 expression (Figure 4C). Next, we wanted to know whether GATA-2 and GATA-3 could down-regulate MICA or MICB expression. We reduced GATA-2 and GATA-3 expression by transfection with GATA-2-siRNA or GATA-3-siRNA into HepG2 cells (Figure 4D) and we found that MICA expression increased significantly at both gene and protein levels after silencing of GATA-2 and GATA-3 (Figure 4E). We then detected the promoter activity of MICA after silencing the expression of GATA-2 and GATA-3. As shown in Figure 4F, silencing GATA-2 and GATA-3 significantly increased the activity of the MICA promoter in HepG2 and HepG2.2.15 cells, suggesting that both of these factors could suppress the transcription of MICA. FACS analysis further confirmed this effect at protein level (Figure 4G). Similarly, GATA-2 and GATA-3 were also shown to depress the transcription and expression of MICB (Supplementary Figure S3C and S3D). These results demonstrated that HBV could augment the expression of GATA-2 and GATA-3, and these two transcription factors further down-regulate the expression of MICA and MICB.

### HBx protein acts as a co-regulator and enhances GATA-2 and GATA-3-mediated suppression of MICA transcription

**Figure 5:**
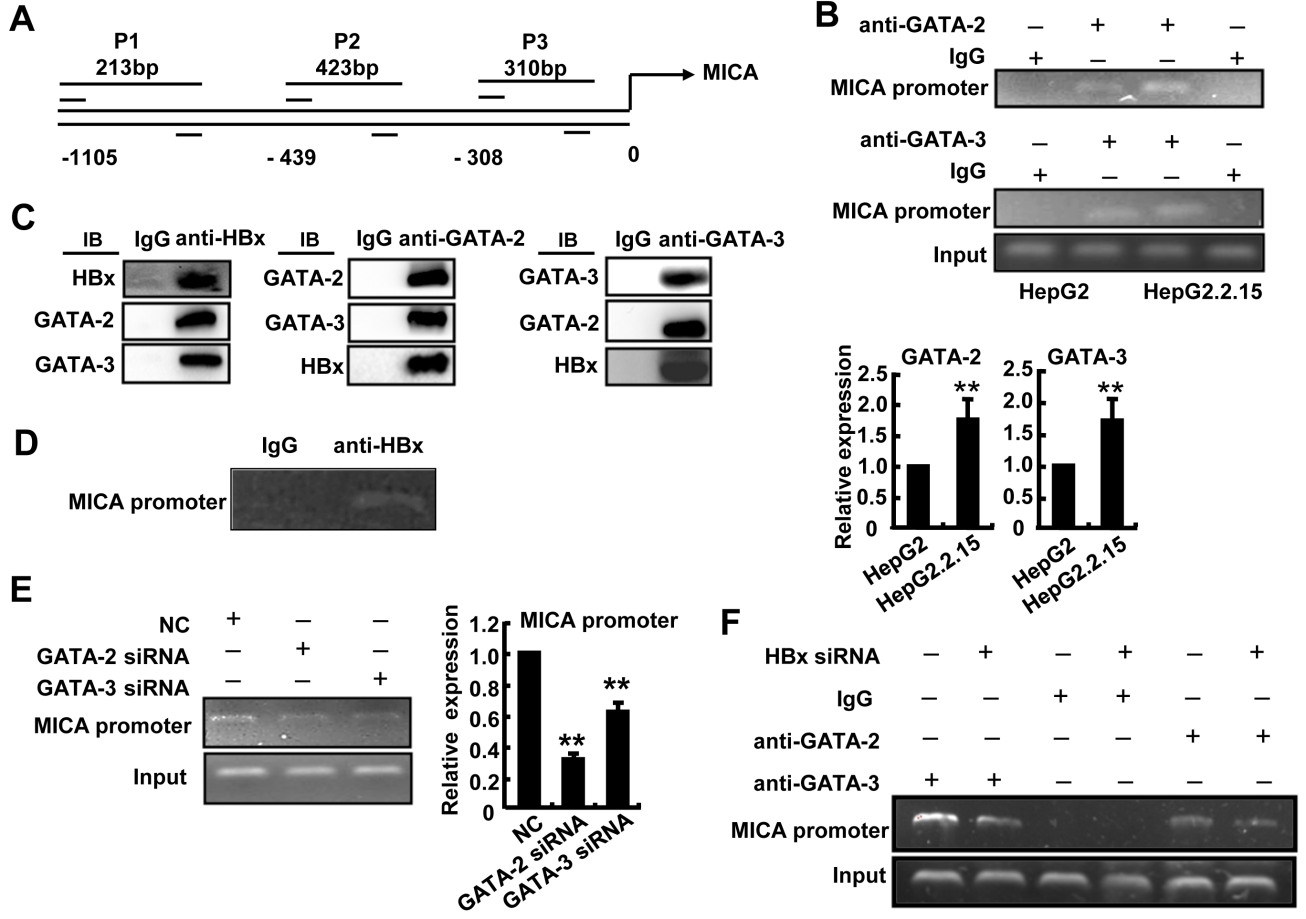
GATA-2 and GATA-3 inhibit MICA transcription, while HBx contributes this effect **A.** Schematic representation of the MICA and MICB gene. Primer sets are indicated as P1, P2, P3. **B.** Soluble chromatin was immunoprecipitated with GATA-2 or GATA-3 antibody. PCR (upper) and qPCR (lower) were used to amplify MICA promoter isolated from the immunoprecipitated chromatin. **C.** Lysates from HepG2.2.15 cells were immunoprecipitated with anti-HBx, anti-GATA-2, anti-GATA-2 or control Ig, and the sample was subjected to Western blotting with indicated antibodies. **D.** A ChIP assay was performed using an anti-HBx antibody or IgG antibody in HepG2.2.15 cells. **E.** GATA-2-siRNA or GATA-3-siRNA was transfected into HepG2.2.15 cells for 48 h, and soluble chromatin was immunoprecipitated with an anti-HBx antibody. PCR (left) and qPCR (right) were used to amplify MICA promoter isolated from the immunoprecipitated chromatin. The experiments were repeated at least three times. **F.** HBx-siRNA was transfected into HepG2.2.15 cells for 48 h, and then soluble chromatin was immunoprecipitated with a GATA-2 or GATA-3 antibody. PCR was used to amplify the MICA promoter isolated from the immunoprecipitated chromatin.

To further confirm the suppression effect of GATA-2 and GATA-3 on MICA promoter activity, a ChIP assay was performed using primers corresponding to −986 (P1), −400 (P2) and −234 (P3) bp upstream of the translation initiation site (ATG) of MICA. Chromatin fragments from HepG2 or HepG2.2.15 were immunoprecipitated with an antibody to GATA-2 or GATA-3. DNA from the immunoprecipitation was isolated, and 213-bp, 423-bp and 310-bp fragments were amplified, respectively, with the P1, P2 and P3 primers of the MICA promoter region. We found that both GATA-2 and GATA-3 could bind to the MICA promoter (P1, P2, P3), and the strongest binding site was at P1 (data not shown). Therefore, we used the P1 primer to amplify the MICA promoter. The PCR analysis revealed a 1.8-fold increase in MICA promoter DNA associated with GATA-2 and a 1.7-fold increase in that associated with GATA-3 in HepG2.2.15 cells compared with HepG2 cells (Figure 5B). The results suggest that HBV promoted the binding of GATA-2 and GATA-3 to the MICA promoter and thus facilitated the down-regulation of MICA expression.

The HBx protein has been reported as a multifunctional regulator and modulates many genes transcription [20]. To explore whether HBx contributes to GATA2 or GATA3-mediated MICA suppression, immunoprecipitation was performed to detect whether HBx can bind to GATA2 or GATA3 and impair their binding ability to MICA promoter in HepG2.2.15 cells. As shown in Figure 5C, GATA-2 and GATA-3 proteins could be detected from complexes immunoprecipitated with the anti-HBx antibody by immunoblot analysis in HepG2.2.15 cells. Also, HBx and GATA3 or GATA2 proteins were detected from complex immunoprecipitated with the anti-GATA2 or anti-GATA3 antibody. These results suggested that HBx could bind to GATA-2 and GATA-3 to form a tripolymer. Furthermore, a 213-bp fragment of the MICA promoter was amplified by PCR with the P1 primer from chromatin fragments of HepG2.2.15 cells immunoprecipitated with an HBx antibody (Figure 5D), suggesting that HBx could indirectly bind to the MICA promoter. Interestingly, after silencing GATA-2 and GATA-3 by respective siRNA, the MICA promoter transcript levels decreased significantly in the anti-HBx immunoprecipitated samples from HepG2.2.15 cells (Figure 5E). Conversely, when HBx expression was reduced by HBx-siRNA, MICA promoter transcript levels immunoprecipitated with the GATA-2 or GATA-3 antibody also decreased significantly (Figure 5F). These results demonstrated that HBx could bind to GATA-2 and GATA-3, thus further enhancing the binding ability of GATA-2 and GATA-3 to MICA promoter. HBx protein might act as a co-regulator contributing to the GATA-2 and GATA-3-mediated down-regulation of MICA expression.

### HBV core protein inhibits MICA/B expression *via* directly binding to the CpG island of MICA/B promoter

**Figure 6:**
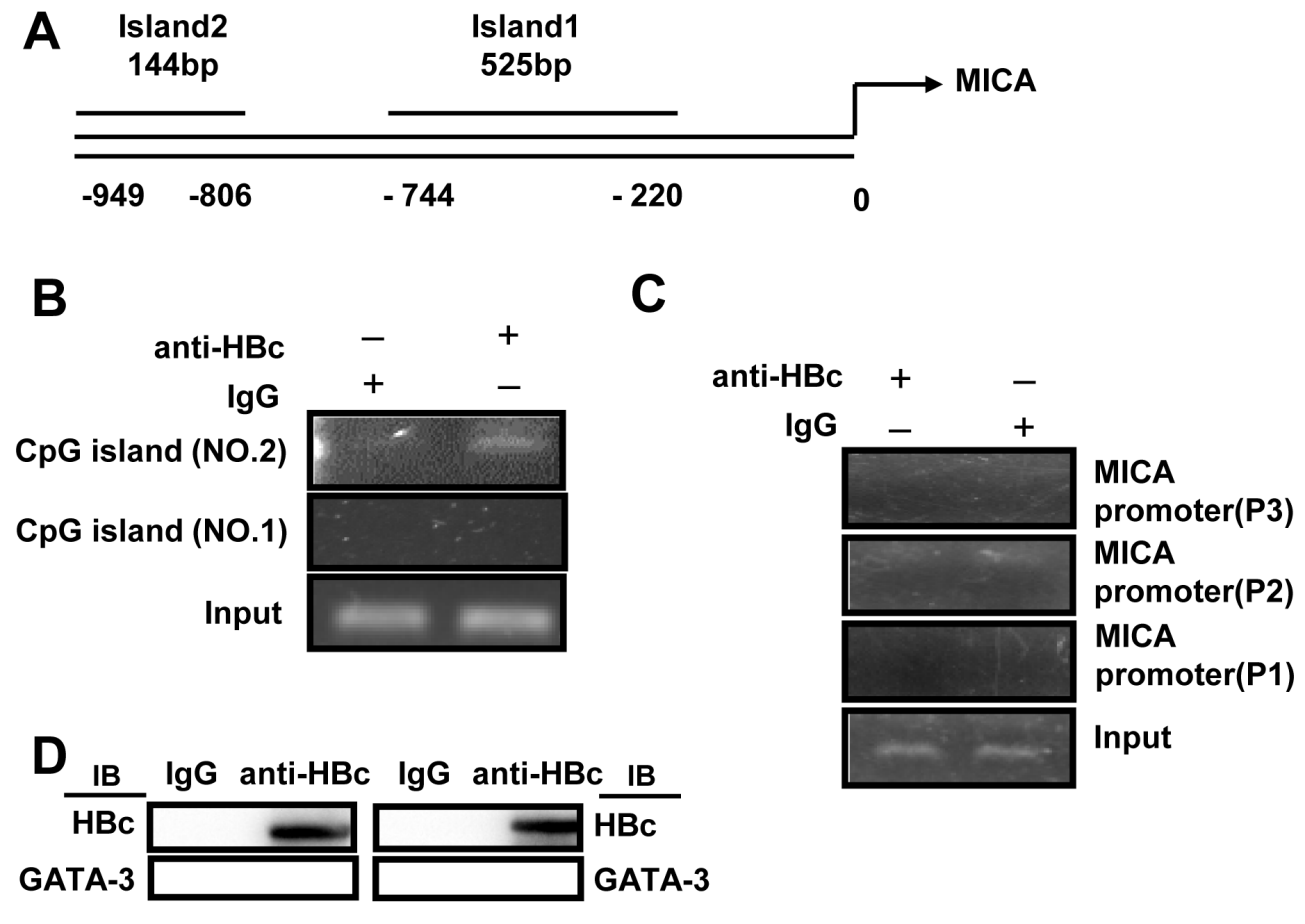
HBV core protein inhibits MICA expression *via* directly binding to the CpG island of MICA promoter **A.** CpG islands were predicted in the MICA promoter. **B.** and **C.** Soluble chromatin was immunoprecipitated with an anti-HBc antibody. PCR was used to amplify the MICA promoter containing CpG island isolated from the immunoprecipitated chromatin. **D.** Lysates from HepG2.2.15 cells were immunoprecipitated with an anti-HBc or control Ig, and then the sample was subjected to Western blotting with indicated antibodies.

Next, we attempted to investigate the role of HBc in the regulation of MICA/B. The HBc protein has been shown to directly bind to promoter regions containing CpG islands [9, 10]. Thus, we predicted two CpG islands in the MICA promoter by using the Emboss cpgplot database (Figure 6A). To determine whether the HBc protein can directly bind with CpG islands in the MICA promoter, chromatin fragments from HepG2.2.15 cells were immunoprecipitated with an anti-HBc antibody. DNA from the immunoprecipitation was isolated, and the two CpG regions were amplified. PCR analysis showed that the HBc protein could bind to CpG island 2 but not CpG island 1 (Figure 6B). In addition, we used the P1, P2 or P3 primer to amplify the MICA promoter with the same DNA from the immunoprecipitation assay, but the MICA promoter was not detected (Figure 6C). Furthermore, the GATA-2 or GATA-3 protein were not be detected from complexes immunoprecipitated with an anti-HBc antibody by immunoblot analysis in HepG2.2.15 cells (Figure 6D). The results indicated that the HBc protein could not bind to the GATA-2 or GATA-3 binding sites. Thus, the HBc protein inhibited MICA expression *via* directly binding to the CpG island 2 of the MICA promoter. As it was shown in Figure S2, HBc also downregulated the expression of MICB, thus, by using the Emboss cpgplot database, we predicted a CpG island in the MICB promoter (Supplementary Figure S4A). ChIP analysis showed that the HBc protein could also bind to CpG island of MICB promoter (Supplementary Figure S4B).

## DISCUSSION

**Figure 7:**
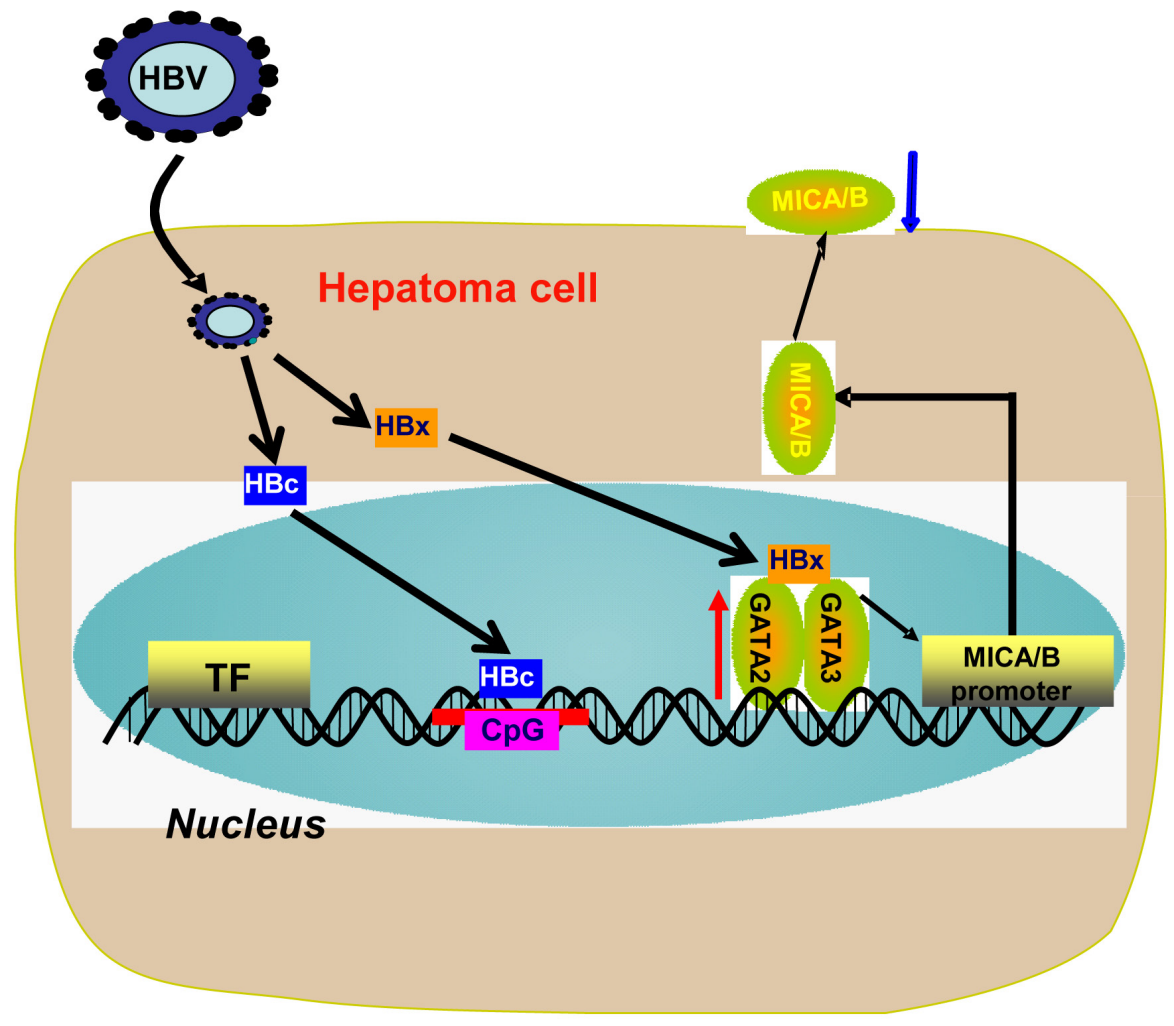
Working model for HBV suppression of MICA/B expression on hepatoma cells Chronic HBV infection up-regulates the expression of transcription factors GATA-2 and GATA-3 in HBV^+^ hepatoma cells. GATA-2 and GATA-3 directly target the MICA/B promoter to inhibit MICA/B transcription. Meanwhile, HBx binds with GATA-2 or GATA-3 and acts as a co-regulator contributing to the GATA-2 and GATA-3-mediated down-regulation of MICA expression. HBc directly binds to the the CpG island of the MICA or MICB promoter and inhibits MICA/B expression.

The precise mechanism for HBV-induced down-regulation of NKG2D ligands on hepatoma cells remains unclear. In the present study, we found for the first time that HBV infection could promote the expression of transcription factors GATA-2 and GATA-3, which specifically suppressed MICA/B expression *via* directly binding to the MICA/B promoter. Moreover, the HBx protein acted as a and contributed to the GATA-2 and GATA-3-mediated suppression of MICA expression. HBc protein could suppress MICA/B expression *via* directly binding to the CpG islands of the MICA or MICB promoter (Figure 7).

NKG2D ligands are not expressed on most normal cells, but they are induced in tumor cells and virus-infected cells. Increasing evidence has shown that cellular stress, infection or tumorigenesis promote the expression of NKG2D ligands [21, 22]. The modulation process may occur at different stages, including transcription, RNA stabilization, protein stabilization and the cleavage from the cell membrane [23]. Several transcription factors, such as heat shock transcription factor 1 (HSF1), NF-κB, Sp1 or Sp3, and STAT3, have been reported to promote the transcription of MICA and MICB by directly binding to their promoter regions [21, 24]. GATA-2 and GATA-3 are members of the GATA family, which contain zinc fingers in their DNA binding domain. GATA-2 is widely regarded as a pivotal regulator for the development and differentiation of hematopoietic stem cells (HSCs) and hematopoietic progenitor cells (HPCs) [18]. GATA-3 has been most extensively studied in T cell development and is regarded as a specific transcription factor for Th2 development [19]. Recently, GATA-2 and GATA-3 were found to be associated with tumorigenesis in various cancers. Overexpression of GATA-2 was detected in a subset of human chronic myelogenous leukemia and human neuroblastoma samples [25, 26], while GATA-3 was shown to be highly expressed in breast cancer, lymphoma and other tumors [27, 28]. Importantly, GATA3 was regarded as a highly breast-specific immunomarker, especially for ER-negative metastatic breast carcinomas, and it was also used to identify a high-risk subset of peripheral T-cell lymphomas [29–31]. However, associations between GATA-2 or GATA-3 with MICA/B expression have not been reported. Here, we demonstrated for the first time that HBV infection enhanced the expression of GATA-2 and GATA-3, which acted as trascriptional repressors and specifically suppressed MICA/B expression. Our results provide a novel mechanism for the role of GATA2 and GATA3 in the escape of HBV^+^ HCC from NK cell immunosurveillance.

The HBx protein is generally known to be important for HBV replication and can regulate cellular transcription, protein degradation, proliferation and apoptotic signaling pathways [32]. It does not bind directly to DNA, its ability to activate transcription involves direct interaction with some transcription factors by protein-protein interactions or stimulation of cytosolic signal-transduction pathways that regulate transcription [8, 33]. Here, we showed for the first time that the HBx protein bound to GATA-2 and GATA-3 to form a tripolymer but not directly binding to the MICA promoter. Such protein-protein interaction between HBx and GATA-2 or GATA-3 strengthened the ability of GATA2/3 binding to the promoter of MICA. These results demonstrated that the HBx protein acted as a co-regulator to promote the GATA-2 or GATA-3-mediated suppression of MICA/B expression, thus further contributing to the escape of HBV^+^ hepatoma from NK lysis. Some reports demonstrated that HBx also transactivates Sp-1 and AP-1 which might enhances MICA expression [34, 35]. In hepatoma cells, athough it seems that HBx-induced GATA3/2 activation and Ap-1 or SP-1 activation might results in opposite effect on MICA expression, we indeed observed that HBx suppresses MICA expression in our experiments. We propose that HBx-transactivated GATA2/3-mediated MICA suppression might be dominant over the up-regulation mediated by SP-1 and AP-1. Indeed, it has been reported that binding activity of SP-1 to the core promoter region of MICA is weaker than other transcription factors [17].

HBc has been reported to bind to the CpG islands of promoters and regulate some gene transcription [9]. In this work, we found two CpG islands in the MICA promoter and one CpG island in the MICB promoter by using the Emboss cpgplot database and further demonstrated that the HBc protein could directly bind to the CpG island in promoter to inhibit MICA/B expression. This finding is the first report of the role and mechanism of the HBc protein in regulation of MICA expression on hepatoma cells, which may contribute to the escape of HBV^+^ hepatoma from NK cell-mediated immunosurveillance.

Due to the critical role of NKG2D and its ligands in the defense against virus infection and tumorigenesis, clarifying how HBV suppresses MICA expression on hepatoma cells and contributes to escape from NK cell lysis is an important line of investigation. HBsAg has been reported to inhibit MICA expression *via* induction of cellular miRNAs in hepatocellular carcinoma cells [36]. The present study suggests that compared with HBsAg, HBx and HBc proteins could more significantly inhibit MICA expression. Furthermore, we identified the novel role of transcription factors GATA-2 and GATA-3 in suppressing MICA/B expression, which contributes to tumor escape from NK lysis. We also illuminated the function of HBx as a co-regulator to promote the GATA-2 or GATA-3-mediated suppression of MICA/B expression and highlighted the direct binding of the HBc protein to CpG island of the MICA/B promoter to down-regulate MICA/B expression. These findings provide novel mechanisms for the contribution of HBV to hepatoma cells escape from NK cell surveillance. Targeted interfering with HBx and HBc may be important therapeutic strategies for HBV^+^ hepatoma and chronic HBV infection.

## MATERIALS AND METHODS

### Cell cultures and reagents

The human HCC cell line HepG2, HepG2.2.15 cells (derived from HepG2 cells transfected with a plasmid carrying two head-to-tail copies of HBV genome DNA serotype ayw), HepG2-HBV cells (derived from HepG2 cells transfected with pEGFP-HBV plasmid and maintain its HBV gene expression by puromycin), human HCC cell lines PLC/PRF/5, H7402 and the human normal hepatocyte cell line HL7702 were maintained in complete DMEM (GIBCO/BRL) supplemented with 10% FBS. The human NK cell line NKL was maintained in RPMI-1640 medium (GIBCO/BRL) containing 10% FBS. All cell lines were incubated at 37°C in a humidified atmosphere with 5% CO_2_. HBx-siRNA was synthesized by Genepharma (Shanghai, China), and GATA-2 and GATA-3 siRNA were purchased from Biosune Corporation (Shanghai, China). The transfection reagent Lipofectamine 2000 was purchased from Invitrogen (Carlsbad, CA, USA), and X-tremeGENE siRNA Transfection Reagent was purchased from Roche (Indianapolis, IN, USA).

### Plasmids and constructions

pEGFP-N1 was purchased from Clontech (Mountain View, CA, USA) and maintained in our lab. To construct plasmids expressing separately the HBx, HBV core protein, HBs and HBV polymerase with green-fluorescent protein (GFP), we amplified the ORF of these four genes from HepG2.2.15 cDNA and inserted them into the pEGFP-N1 vector. The pEGFP-HBV plasmid was constructed by inserting the full-length HBV DNA into the pEGFP-N1 vector. The pRL-TK and pGL3-basic plasmids were purchased from Promega (Hong Kong). pGL3-MICA, MICB promoter and HBc-shRNA were constructed by our lab.

### RNA isolation and quantitative PCR (qPCR)

Total RNA was isolated using the TRIZOL reagent (Invitrogen) according to manufacturer's instructions. cDNA was obtained by using oligo (dT) primer, dNTP and M-MLV Reverse Transcriptase (Invitrogen) as instructed. Real-time qPCR was performed using the Roche SYBR Green mix. The primers are described in Supplementary Table S1.

### Luciferase reporter gene assay

The 3′-UTR of the human MICA cDNA containing the putative target site for GATA-2 and GATA-3 was inserted into the pMIR-Reporter-control vector (Promega, Madison, WI, USA) immediately downstream of the luciferase gene. HepG2 or HepG2.2.15 cells were transfected with PGL3-basic, PGL3-MICA promoter or PGL3-MICB promoter together with pRL-TK using Lipofectamine 2000 according to the manufacturer's protocol. Luciferase activity was measured at 36 h after transfection using the Dual Luciferase Reporter Assay System (Promega). Firefly luciferase activity was normalized to Renilla luciferase activity for each transfected well. Three independent experiments were performed in triplicate.

### Flow cytometry

Surface staining was performed using the following anti-mouse monoclonal antibodies (mAbs) or antibody controls: PE-conjugated IgG isotype and anti-MICA/B, anti-MICA, anti-MICB, anti-ULBP1, anti-ULBP2, anti-ULBP3, anti-HLA-ABC mAb (R&D Systems, Minneapolis, MN, USA), anti-PD-L1 mAb (eBioscience, San Diego, CA, USA), Percp-cy5.5-conjugated IgG isotype and anti-CD95 mAb . Cells were collected with

### Western blot analysis

Proteins from cell lysates in loading buffer were resolved on 10% SDS polyacrylamide gels. The rabbit anti-MICA mAb was purchased from Epitomics (Burlingame, CA, USA). Rabbit anti-human GATA-2 mAb was purchased from Abcam (Cambridge, UK). Rabbit anti-GFP mAb (Abcam), Rabbit anti-human GATA-3 mAb (eBioscience), and mouse anti-human β-actin was purchased from Santa Cruz Biotechnology (Santa Cruz, CA, USA). Protein bands were observed by enhanced chemiluminescence (Millipore, Bedford, MA, USA) and were determined by using ImageLab software (version 3.0, Bio-Rad Laboratories, Hercules, CA, USA).

### Cytotoxicity assay

NK cell cytotoxicity against hepatocellular carcinoma cells and blocking experiments were assessed by the CFSE/7AAD method. For the CFSE/7AAD method, target cells were labeled with CFSE, and then the target and effector cells were analyzed by using a MICA/B neutralizing antibody was purchased from R&D Systems.

### Chromatin immunoprecipitation (ChIP) assay

HepG2 and HepG2.2.15 cells were plated at a density of 1 × 10^6^ cells in 10-cm dishes. The ChIP assay was performed using the EZ ChIP kit (Millipore). Sequences of the PCR primers of MICA and MICB promoter are described in Supplementary Table S2. Rabbit anti-human GATA-2 mAb (ChIP grade, Abcam), rabbit anti-human GATA-3 mAb (eBioscience), mouse anti-human β-actin mAb (Santa Cruz Biotechnology), anti-mouse IgG and anti-rabbit IgG were (Jinqiao, Beijing, China) were obtained commercially.

### Immunoprecipitation assay

Cells were harvested by scraping in an immunoprecipitation assay buffer (25 mM Tris, 150 mM NaCl, 1 mM EDTA, 1 mM EGTA, 20 mM sodium molybdate, and 0.5% Nonidet P-40) containing phosphatase and proteinase inhibitors (BestBioscience, Shanghai China). Lysates were clarified for 30 min at 13,000 × *g*, 4°C. A portion of the supernatants was incubated sequentially at 4°C overnight with protein A (Millipore) conjugated to an anti-HBx antibody (Abcam) or anti-HBc antibody (Abcam). Magnetic beads were washed three times with 200 μl of ice-cold immunoprecipitation assay buffer. Bound protein complexes and input fractions were examined by Western blot analysis using anti-human GATA-2 (Abcam) or anti-human GATA-3 (eBioscience) antibodies.

### Statistical analysis

Statistical analysis was performed using a paired Student's *t*-test. Differences were considered significant at *P* < 0.05.

## SUPPLEMENTARY MATERIAL FIGURE AND TABLES


