# Supplementary material for: HBV suppresses expression of MICA/B on hepatoma cells through up-regulation of transcription factors GATA2 and GATA3 to escape from NK cell surveillance
Source: Oncotarget. 2016 Aug 12;7(35):56107–19. doi: 10.18632/oncotarget.11271 (PMC5302899; doi:10.18632/oncotarget.11271)
Supplement: Supplementary file 1 [file oncotarget-07-56107-s001.pdf]

HBV suppresses expression of MICA/B on hepatoma cells through up-regulation of transcription factors GATA2 and GATA3 to escape from NK cell surveillance

Supplementary Material

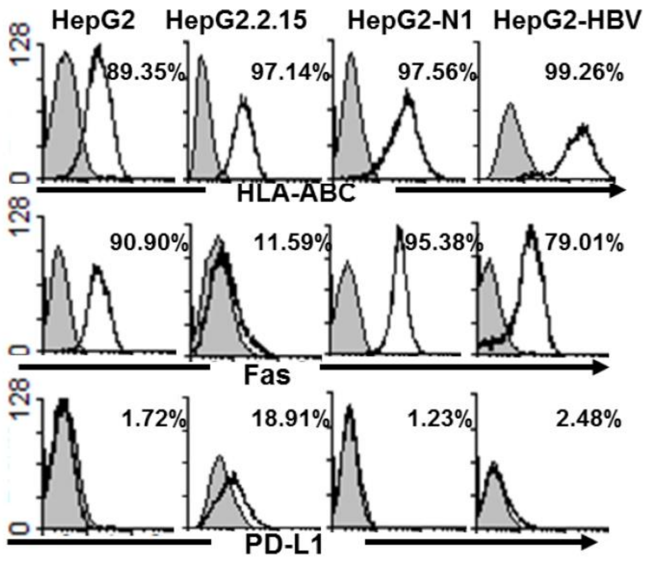

Supplementary Figure 1. HBV^{+} hepatoma cells express different levels of HLA-ABC, Fas and PD-L1 compared with HBV^{+} hepatoma cells. Expression levels of HLA-ABC, Fas and PD-L1 on HepG2, HepG2.2.15, HepG2-N1 and HepG2-HBV cells were analyzed by FACS.

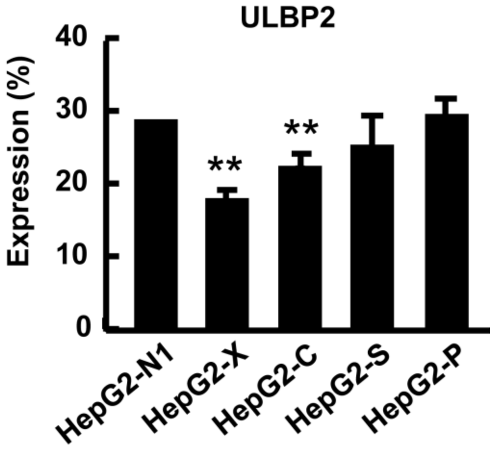

Supplementary Figure 2. HBx and HBc genes suppress ULBP2 expression in hepatoma cells. pEGFP-N1, pEGFP-HBx, pEGFP-HBc, pEGFP-HBs or pEGFP-HBp was transfected into HepG2 cells for 48 h. ULBP2 was analyzed by FACS. **$P < 0.01$, compared with HepG2-N1 (paired t-test).

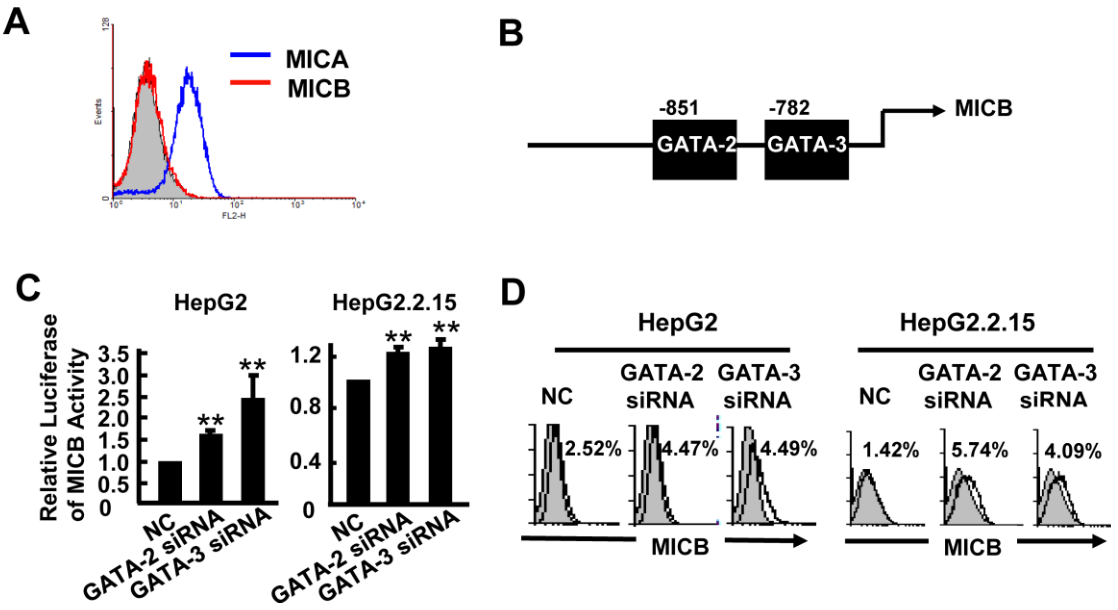

Supplementary Figure 3. Transcription factors GATA-2 and GATA-3 are involved in regulation of MICB expression. (A) Flow cytometric analysis of MICA and MICB expression in HepG2 cells. (B) Prediction of GATA-2 and GATA-3 binding sites in the MICB promoter. (C) HepG2 cells or HepG2.2.15 cells were transfected with GATA-2-siRNA or GATA-3-siRNA together with the MICB promoter for 36 h. Firefly luciferase activity was normalized to renilla luciferase activity. (D) Flow cytometric analysis of MICB expression in HepG2 or HepG2.2.15 cells after transfection with GATA-2-siRNA or GATA-3-siRNA. $^{**}P < 0.01$, compared with negative control (paired t-test).

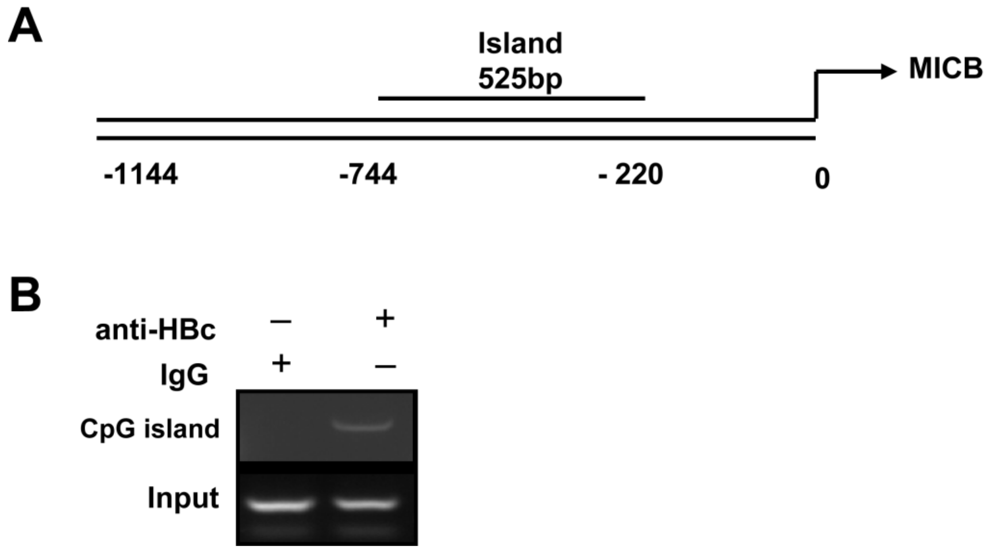

Supplementary Figure 4. HBV core protein inhibits MICB expression via directly binding to the CpG island of MICB promoter. （A）CpG island were predicted in the MICB promoter. (B) Soluble chromatin was immunoprecipitated with an anti-HBc antibody. PCR was used to amplify the MICA promoter containing the CpG island isolated from the immunoprecipitated chromatin.

Supplementary Tables

Table S1. Sequences of primers used for semi-quantitative PCR assay

GAPDH	F: 5'-GAAGGTGAAGGTCGGAGT-3<br>R: 5'-CATGGGTGGAATCATATTGGAA-3'
GATA-2	F: 5'-ACTGACGGAGAGCATGAAGAT-3'<br>R: 5'-CCGGCACATAGGAGGGGTA-3'
GATA-3	F: 5'-GCCCCTCATTAAGCCCAAG-3'<br>R: 5'-TTGTGGTGGTCTGACAGTTCG-3'
MICA	F: 5'-GAAGGTGAAGGTCGGAGT-3'<br>R: 5'-CATGGGTGGAATCATATTGGAA-3'
MICB	F: 5'-GAAGGTGAAGGTCGGAGT-3'<br>R: 5'-CATGGGTGGAATCATATTGGAA-3'

Table S2. Sequences of the PCR primers of promoters for ChIP assay

MICA (P1)	F: 5'-CTGTGGGATTGAAATAGCG-3'<br>R: 5'-CAGCCTGCCTTAGGACAT-3'
MICA (P2)	F: 5'-GGAAGGAACAAGCCAGTG-3'<br>R: 5'-GCCAGAAGCAGGAAGACC-3'
MICA (P3)	F: 5'-CCGTGCTTATGAAGTTGG-3'<br>R: 5'-AGCCAGAAGCAGGAAGAC-3'
MICB	F: 5'-TCTCCAGGCCGCTAGAAT-3'<br>R: 5'-AGGAACCCCACTCACCAG-3'
MICA CpG Island 1	F: 5'- GGGAAAGAGGAAATCAGAR-3'<br>R: 5'-CGTTACTGGCAATGAGAC-3'
MICA CpG Island 2	F: 5'-CTGGTGGGATAGGGTGAG-3'<br>R: 5'-CATCCTTGGTCCCTTCTG-3'
MICB CpG Island	F: 5'-TGGGAGAAAAACCACGCGTT-3'<br>R: 5'- GGTGCAAAAGGGAAGGCGA-3'
